# Why the Therapeutic Impact of RAS Mutation Clearance in Plasma ctDNA Deserves to Be Further Explored in Metastatic Colorectal Cancer

**DOI:** 10.3389/fonc.2019.01414

**Published:** 2019-12-17

**Authors:** Chiara Nicolazzo, Francesca Belardinilli, Salvatore Caponnetto, Angela Gradilone, Enrico Cortesi, Giuseppe Giannini, Paola Gazzaniga

**Affiliations:** ^1^Liquid Biopsy Unit, Department of Molecular Medicine, Sapienza University of Rome, Rome, Italy; ^2^Department of Molecular Medicine, Sapienza University of Rome, Rome, Italy; ^3^Department of Radiological, Oncological and Pathological Sciences, Sapienza University of Rome, Rome, Italy; ^4^Institute Pasteur-Cenci Bolognetti Foundation, Rome, Italy

**Keywords:** ctDNA, RAS, EGFR inhibitiors, liquid biopsy, colorectal cancer

## Introduction

An increasingly large body of evidence supports the assertion that the analysis of circulating tumor DNA (ctDNA) in metastatic colorectal cancer (mCRC) patients allows the temporal heterogeneity of cancer during the course of targeted therapies to be monitored. From liquid biopsy-guided genomic studies in mCRC, we have learned that there may be a rise in RAS mutant clones in the plasma of patients before the onset of secondary resistance to anti-EGFR therapy, generating new hypotheses for blood-guided adaptive therapeutic strategies (1). Similarly, the clearance of RAS mutant clones in plasma has been more recently suggested, supporting, for the first time, an unexpected negative selection of RAS mutant clones during the clonal evolution of mCRC (2–4). This phenomenon had previously been described in leukemia patients, with some showing a loss of RAS mutations during disease progression, supporting the hypothesis that the evolutionary pressure of therapies can result in positive but also negative selection of RAS mutant clones at relapse (5). The temporary prevalence of wt RAS clones at relapse in mCRC raises the question of whether liquid biopsy testing might expand the population of anti-EGFR-eligible patients by including those with primary RAS-mutant mCRC that “convert” to wild type in plasma at the time of disease progression (PD). Whether undetectable RAS mutations in plasma might really reflect wt RAS status or might simply mirror a low analytic sensitivity in the adopted assays is still a matter of debate (6).

## Clearance of RAS Mutation in Plasma: Tumor-Specific Mutations to Confirm/Exclude the Presence of ctDNA

It is undeniable that correct interpretation of the clearance of RAS mutations in plasma must be supported by the demonstration of detectable ctDNA; therefore, the crux of the matter is the urgent need for appropriate methods to confirm (or to exclude) the presence of ctDNA in plasma samples. For this purpose, plausible options would be (1) to monitor a somatic mutation other than RAS that was previously detected in the primary tissue, (2) to provide evidence of wt RAS status in metastatic sites at the time of PD. To date, two groups have investigated the clearance of RAS mutations in plasma at the time of PD for therapeutic purposes in patients with primary RAS-mutant mCRC, even if in small sample series. The first study reported a clinical benefit achieved by some patients with RAS-mutant primary mCRC treated with anti-EGFR, based on detection of wt RAS status in plasma at the time of PD (2). In this series, 11 patients were screened via liquid biopsy at PD, and in 5/11 cases (45%) no RAS mutation was detected in plasma. Unfortunately, for only one patient did the authors provide evidence that the disappearance of RAS mutation was concomitantly associated with the persistence of one somatic mutation previously detected in the primary tumor. In the other four cases, the absence of any somatic mutation other than RAS that could be monitored in the blood led to “inconclusive” results. Globally, the real “RAS clearance rate,” calculated after excluding the inconclusive samples from the whole population, was 1/7 (14%). Owing to these preliminary data, a phase II prospective study was planned (7, 8) with the aim of investigating whether targeting the plasma wt RAS window with EGFR-inhibitors might represent on a large scale an exploitable second-line option in RAS-mutant mCRC. In 2019, a similar phase II prospective trial was aimed at evaluating the efficacy and safety of FOLFIRI plus panitumumab as a second-line treatment in mCRC patients with RAS mutant primary mCRC with no evidence of RAS mutation in plasma at the time of PD (9). Preliminary results have shown that of the 16 patients analyzed, only two had wt RAS mCRC after first-line treatment. Both were screen failures, so no patient has been included to date (9).

## Clearance of RAS Mutation in Plasma: Methylation to Confirm/Exclude the Presence of ctDNA

The detection of tumor-specific DNA methylation alterations in ctDNA has recently been suggested as a specific tool for confirming the tumoral origin of ctDNA. Moati et al. (4) investigated the clearance of RAS mutant clones under chemotherapy pressure by ctDNA analysis in patients with RAS mutant mCRC. By monitoring ctDNA with methylated markers (WIF1 and NPY genes), the authors concluded that the clearance of RAS mutations by chemotherapy is actually a rare event. Moati et al. define the clearance of RAS mutation as “*the disappearance of RAS mutation concomitantly associated with the persistence of ctDNA proven by the detection of either (i) at least one mutation in other genes by NGS, (ii) or methylation of WIF1 and NPY genes determined by ddPCR (met-ddPCR*).” All samples with no mutation and no WIF1/NPY methylation were considered as “inconclusive.” In their study, Moati et al. described 8/36 (22%) patients who converted to a wt-RAS status in blood at the time of disease progression, but only in two patients was the presence of ctDNA in the sample confirmed by methylation test, while for the other six cases, the results were inconclusive. Thus, taking samples with inconclusive results out of the whole series, Moati et al. found a real switch from mutant to wt-RAS at progression in 2/30 patients (6.6%). Although an increasing amount of evidence has been provided that DNA methylation markers may be employed to track response during therapy in mCRC (10), the use of a WIF-1/NPY-specific methylation test to confirm the presence of ctDNA unfortunately relies on two proof of concept/exploratory studies, strongly impairing the generalization of their conclusions. Specifically, these two studies have evaluated WIF-1/NPY methylation dynamics in mCRC cases on treatment: (1) Garrigou et al. (11), who failed to detect methylation by liquid biopsy in 20% of mCRC, and (2) Garlan et al. (12), who, using the same test, showed only 69.2% positivity in KRAS/BRAF/TP53 wild-type mCRC. A more recent study (13), using high genome-coverage methods, demonstrated that other methylated loci are more compliant to liquid biopsy analyses, suggesting that their use in combination might improve the positivity of ctDNA detection. In this study, *NPY* was discarded due to positivity in normal mucosa and in whole blood samples (which contain white blood cells as the main contaminant of cfDNA), and *WIF1* was found to have limited differential methylation between normal healthy/normal adjacent mucosa and tumor. Other recently published studies found NPY methylation in the plasma of healthy donors (14) and non-cancer patients (15). Therefore, to date, we are far from true agreement on the use of WIF-1/NPY methylation testing to exclude or confirm the presence of ctDNA. Table 1 is a summary of the studies that have to date investigated the clearance rate of RAS mutations in plasma at the time of disease progression in patients with metastatic colorectal cancer.

**Table 1:** Studies that investigated the clearance of RAS mutations in the plasma of metastatic colorectal cancer patients at the time of progression of disease.

**References**	***N* patients**	**ctDNA assay used**	**Patients with clearance of RAS mutations in plasma at PD (%)**	**Patients with confirmed ctDNA presence in plasma** **at PD (%)**	**Method to confirm** **ctDNA presence**
Raimondi et al. (3)	11	Idylla	5/11 (45%)	1/7 (14%)	NGS
Moati et al. (4)	36	ddPCR/NGS	8/36 (22%)	2/30 (6.6%)	Methylation test
Fernández Montes et al. (9)	15	Not specified	2/15 (13%)	2/15 (13%)	Not specified

## Discussion

To date, few studies have reported the clearance of RAS mutations in the plasma of mCRC patients. Comparing these studies, the clearance rate of RAS mutation was globally higher in Raimondi's series, which could be consistent with the lower sensitivity of the method used (Idylla^TM^, Biocartis), although no definitive conclusions can be drawn due to the small sample size of both studies. In this context, a recent comparison between the OncoBEAM RAS CRC mutation test and Idylla ctKRAS assay from paired plasma samples of mCRC patients identified a “gray zone” below a 1% mutant allele fraction (MAF) where Idylla shows reduced RAS mutation detection accuracy vs. OncoBEAM (16). On the other hand, an increased sensitivity of RAS testing under 1%, which is undoubtedly useful for monitoring the early onset of mutations, in this specific case might risk excluding from treatment patients with low MAF, who might benefit from anti-EGFR (17). In this respect, the use of an excessively sensitive method to detect RAS mutations in blood could represent one plausible explanation for the missed enrollment of patients in the Convertix trial. To sum up, our opinion is that the collective effort must be aimed at the search for one or more methods that allow the presence of ctDNA in plasma samples to be confirmed, thus decreasing the unacceptably high percentage of “inconclusive” results. Possibly, the use of NGS panels covering a broader spectrum of mutations (better if specific for colorectal cancer) will improve the detection rate of somatic mutations in tumor tissues to be longitudinally monitored in plasma. Nevertheless, the few datasets that are available suggest that the percentage of patients with proven RAS mutation clearance (14 and 6.6% in the Italian and French study, respectively) is such as to indicate that the therapeutic implication of these findings merit being further addressed. Lessons from HER-2-amplified and MSI-H mCRC, which represent 4 and 5% of mCRC, respectively (18), should be a general warning that if we can treat a few patients, or even one patient, then personalized medicine will have achieved its main purpose.

## Author Contributions

All authors listed have made a substantial, direct and intellectual contribution to the work, and approved it for publication.

### Conflict of Interest

The authors declare that the research was conducted in the absence of any commercial or financial relationships that could be construed as a potential conflict of interest.

## Abbreviations

ctDNA: circulating tumor DNA
ddPCR: droplet digital PCR
mCRC: metastatic colorectal cancer
NGS: next-generation sequencing
wt: wild-type
PD: progression of disease.

## References

[1] Siravegna G Mussolin B Venesio T Marsoni S Seoane J Dive C. How liquid biopsies can change clinical practice in oncology. Ann Oncol. (2019) 30:1580–90. 10.1093/annonc/mdz227 31373349

[2] Gazzaniga P Raimondi C Urbano F Cortesi E EGFR inhibitor as second-line therapy in a patient with mutant RAS metastatic colorectal cancer: circulating tumor DNA to personalize treatment. JCO Precis Oncol. (2018) 2:1–6. 10.1200/PO.17.00277 35135115

[3] Raimondi C Nicolazzo C Belardinilli F Loreni F Gradilone A Mahdavian Y. Transient disappearance of RAS mutant clones in plasma: a counterintuitive clinical use of EGFR inhibitors in RAS mutant metastatic colorectal cancer. Cancers. (2019) 11:E42. 10.3390/cancers11010042 30621206 PMC6357143

[4] Moati E Blons H Taly V Garlan F Wang-Renault SF Pietrasz D. Plasma clearance of RAS mutation under therapeutic pressure is a rare event in metastatic colorectal cancer. Int J Cancer. (2019). 10.1002/ijc.32657 31472013

[5] Ma X Edmonson M Yergeau D Muzny DM Hampton OA Rusch M. Rise and fall of subclones from diagnosis to relapse in pediatric B-acute lymphoblastic leukaemia. Nat Commun. (2015) 6:6604. 10.1038/ncomms7604 25790293 PMC4377644

[6] Antoniotti C Pietrantonio F Corallo S de Braud F Falcone A Cremolini C Circulating tumor DNA analysis in colorectal cancer: from dream to reality. JCO Precis Oncol. (2019) 3:1–14. 10.1200/PO.18.00397 35100685

[7] Gazzaniga P Raimondi C Nicolazzo C Gradilone A Cortesi E ctDNA might expand therapeutic options for second line treatment of KRAS mutant mCRC. Ann Oncol. (2017) 28(Suppl. 5):v573–94. 10.1093/annonc/mdx390

[8] Gazzaniga P Raimondi C Urbano F Cortesi E Second line EGFR-inhibitors in RAS mutant metastatic colorectal cancer: the plasma RAS wild type window of opportunity. Ann Oncol. (2018) 29(Suppl. 8):viii150–204. 10.1093/annonc/mdy281

[9] Fernández Montes A Martinez Lago D De la Cámara Gómez J Covela Rúa M Cousillas Castiñeiras A Gonzalez Villarroel P FOLFIRI plus panitumumab as second-line treatment in mutated RAS metastatic colorectal cancer patients who converted to wild type RAS after receiving first-line FOLFOX/CAPOX plus bevacizumab-based treatment: phase II CONVERTIX trial. Ann Oncol. (2019) 30 10.1093/annonc/mdz155.088

[10] Ma Z Williams M Cheng YY Leung WK. Roles of methylated DNA biomarkers in patients with colorectal cancer. Dis Markers. (2019) 2019:2673543. 10.1155/2019/2673543 30944663 PMC6421784

[11] Garrigou S Perkins G Garlan F Normand C Didelot A Le Corre D. A study of hypermethylated circulating tumor DNA as a universal colorectal cancer biomarker. Clin Chem. (2016) 62:1129–39. 10.1373/clinchem.2015.253609 27251038

[12] Garlan F Laurent-Puig P Sefrioui D Siauve N Didelot A Sarafan-Vasseur N. Early evaluation of circulating tumor DNA as marker of therapeutic efficacy in metastatic colorectal cancer patients (PLACOL study). Clin Cancer Res. (2017) 23:5416–25. 10.1158/1078-0432.CCR-16-3155 28576867

[13] Barault L Amatu A Siravegna G Ponzetti A Moran S Cassingena A. Discovery of methylated circulating DNA biomarkers for comprehensive non-invasive monitoring of treatment response in metastatic colorectal cancer. Gut. (2018) 67:1995–2005. 10.1136/gutjnl-2016-313372 28982739 PMC5897187

[14] Thomsen CB Andersen RF Lindebjerg J Hansen TF Jensen LH Jakobsen A Correlation between tumor-specific mutated and methylated DNA in colorectal cancer. JCO Precis Oncol. (2019) 3:1–8. 10.1200/PO.18.00162 35100675

[15] Crujeiras AB Campion J Díaz-Lagares A Milagro FI Goyenechea E Abete I. Association of weight regain with specific methylation levels in the NPY and POMC promoters in leukocytes of obese men: a translational study. Regul Pept. (2013) 186:1–6. 10.1016/j.regpep.2013.06.012 23831408

[16] Vivancos A Aranda E Benavides M Élez E Gómez-España MA Toledano M. Comparison of the clinical sensitivity of the Idylla platform and the OncoBEAM RAS CRC assay for KRAS mutation detection in liquid biopsy samples. Sci Rep. (2019) 9:8976. 10.1038/s41598-019-45616-y 31222012 PMC6586620

[17] Dienstmann R Salazar R Tabernero J. Molecular subtypes and the evolution of treatment decisions in metastatic colorectal cancer. Am Soc Clin Oncol Educ. Book. (2018) 38:231–8. 10.1200/EDBK_200929 30231342

[18] Sandhu J Lavingia V Fakih M. Systemic treatment for metastatic colorectal cancer in the era of precision medicine. J Surg Oncol. (2019) 119:564–82. 10.1002/jso.25421 30802315

